# Genetic and histopathological analysis of a case of primary intraosseous carcinoma, NOS with features of both ameloblastic carcinoma and squamous cell carcinoma

**DOI:** 10.1186/s12957-020-01827-6

**Published:** 2020-02-29

**Authors:** Akane Yukimori, Maiko Tsuchiya, Akane Wada, Yasuyuki Michi, Kou Kayamori, Kei Sakamoto, Tohru Ikeda

**Affiliations:** 1 Department of Diagnostic Oral Pathology, Graduate School of Medical and Dental Sciences, Tokyo Medical and Dental University, 1-5-45 Yushima, Bunkyo-ku, Tokyo, 113-8549 Japan; 2 Present Address: Department of Oral Pathology, School of Dentistry, Showa University, 1-5-8 Hatanodai, Shinagawa-ku, Tokyo, 142-8555 Japan; 3 Department of Oral Pathology, Graduate School of Medical and Dental Sciences, Tokyo Medical and Dental University, 1-5-45 Yushima, Bunkyo-ku, Tokyo, 113-8549 Japan; 4 Department of Oral and Maxillofacial Surgery, Graduate School of Medical and Dental Sciences, Tokyo Medical and Dental University, 1-5-45 Yushima, Bunkyo-ku, Tokyo, 113-8549 Japan

**Keywords:** Ameloblastoma, Intraosseous carcinoma, Malignant tumor, Next-generation sequencing, Odontogenic carcinoma

## Abstract

**Background:**

Primary intraosseous carcinoma (PIOC), NOS is an odontogenic carcinoma with unknown etiology. Its diagnosis may be used when central jaw carcinoma cannot be categorized as any other type of carcinoma. Further information on this extremely rare tumor is needed to improve our understanding and evaluate the classification of odontogenic carcinomas.

**Case presentation:**

We herein presented two patients with PIOC, NOS with different clinical and histopathological features and analyzed gene mutations in these patients using next-generation sequencing (NGS). The typical PIOC, NOS case had many histopathological similarities to oral squamous cell carcinoma (OSCC), including the missense point mutations of *TP53 Glu285Val*, *KDR Gln472His*, and *APC Pro1433Leu*, which are similar to those in other cancers; however, no mutations were detected in the other patient with an atypical presentation of PIOC, NOS, which was derived from a precursor cystic lesion with similarities to both ameloblastic carcinoma and OSCC.

**Conclusions:**

Genetic analysis suggested that these two PIOC, NOS cases have different features and can be subcategorized.

## Background

As described in the WHO Classification of Head and Neck Tumors, primary intraosseous carcinoma (PIOC), NOS is a central jaw carcinoma that cannot be categorized as any other type of carcinoma. PIOC, NOS is assumed to arise from odontogenic epithelium. Some cases arise from odontogenic cysts or other benign precursors. Most lesions are squamous and composed of islands or small nests of a neoplastic squamous epithelium [1]. This type of tumor was described as primary intraosseous squamous cell carcinoma in previous editions of the WHO classification and was further classified into three subtypes: solid type, derived from keratocystic odontogenic tumors, and derived from odontogenic cysts [2]. The previous classification strongly suggested that primary intraosseous squamous cell carcinoma comprises intragnathic tumors derived from multiple origins, and genetic information needs to be applied in addition to histopathological findings to evaluate the classification of PIOC, NOS.

Recent studies using next-generation sequencing (NGS) revealed several gene mutations in odontogenic tumors, including ameloblastoma [3]; however, mutations in PIOC, NOS remain unclear because of the low incidence of this tumor. We herein report two patients with PIOC, NOS with varying histopathological features. We also analyzed genetic mutations in these patients using NGS and compared their genetic and histopathological features.

## Case presentation

### Case 1

**Fig. 1:**
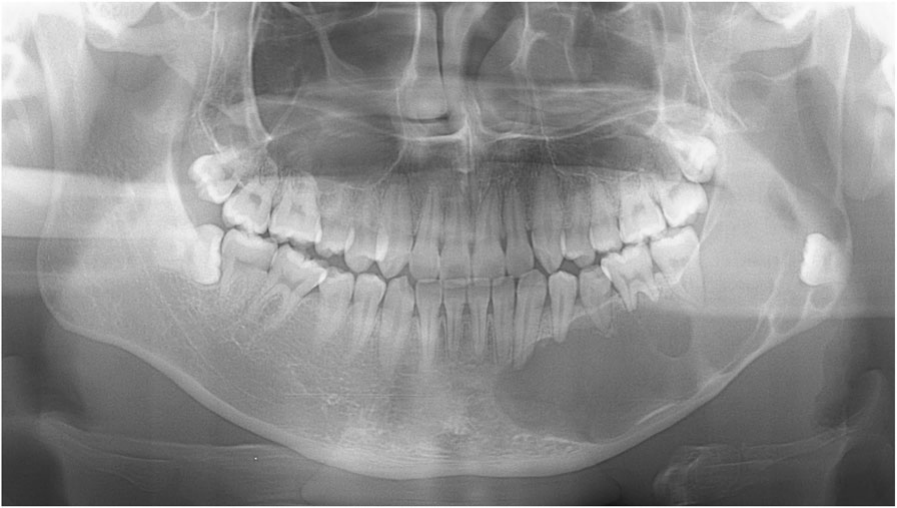
Panorama X-ray photograph of case 1 at the first visit. Multilocular radiolucent lesions in the left mandibular region

**Fig. 2:**
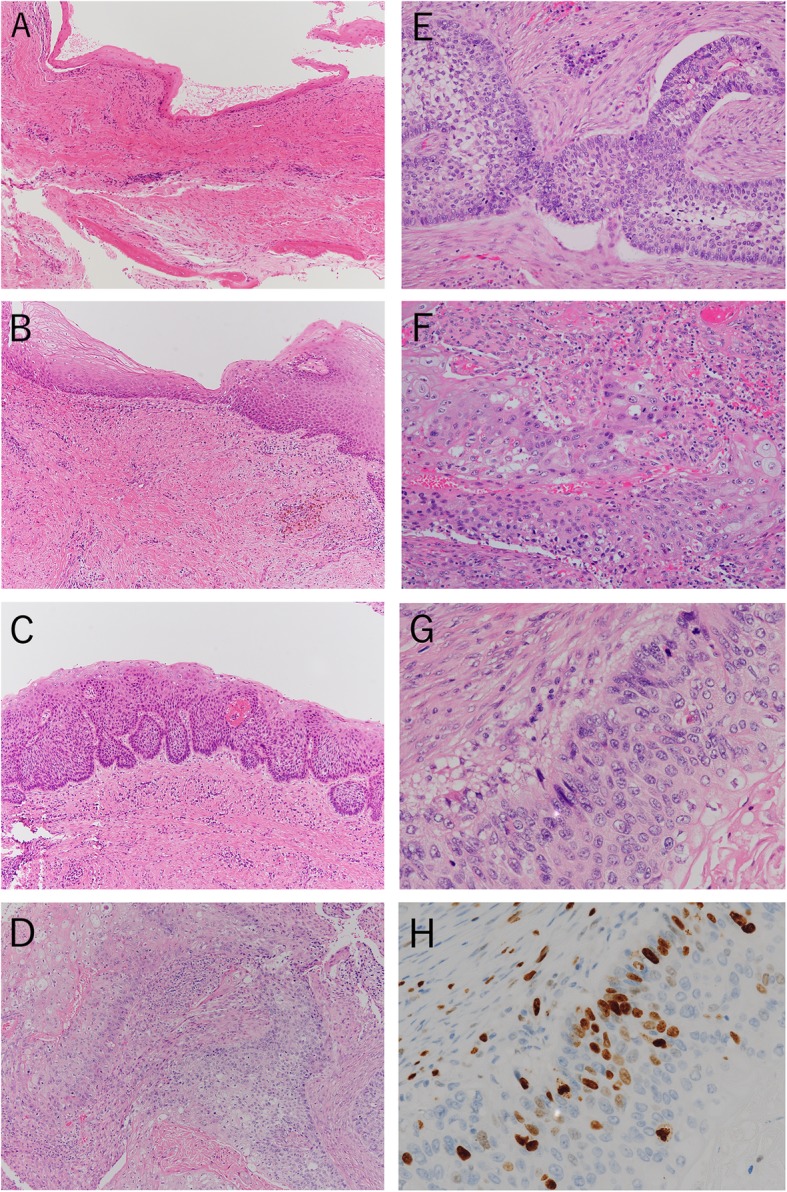
The transition of histopathological findings of case 1. **a** A cystic lesion in the biopsy specimen at the first visit of the patient (hematoxylin & eosin [H&E] staining, original magnification × 100). **b** A cystic lesion in the enucleated specimen after fenestration (H&E staining, original magnification × 100). **c** A cystic lesion in the enucleated specimen with ameloblastoma-like findings after fenestration (H&E staining, original magnification × 100). **d** Tumor infiltration in the enucleated specimen of the recurrent lesion (H&E staining, original magnification × 100). **e** An ameloblastoma-like lesion in the enucleated specimen of the recurrent lesion (H&E staining, original magnification × 200). **f** An OSCC-like lesion in the enucleated specimen of the recurrent lesion (H&E staining, original magnification × 200). **g** High-power magnification of the OSCC-like lesion in the enucleated specimen of the recurrent lesion. The specimen was composed of atypical tumor cells with mitoses (H&E staining, original magnification × 400). **h** Immunohistochemistry of the tumor using Ki-67 (original magnification × 400)

A 28-year-old Japanese male presented with a swelling of his left mandible visited our hospital. He was a nonsmoker and had dull pain in the left molar region of the mandible. A clinical examination revealed slight facial asymmetry and an elastic hard mass in the left molar region. The presence of a multilocular radiolucent lesion was observed in the left lateral incisor to the mandibular condyle area (Fig. 1), and the clinical diagnosis was an ameloblastoma or a keratocystic odontogenic tumor (current name for an odontogenic keratocyst). A biopsy was performed, and the histopathological diagnosis was a cystic lesion suggestive of a keratocystic odontogenic tumor (Fig. 2a). After fenestration, the patient was assessed to evaluate the prognosis of the condition at 1 year and 3 months, and surgical enucleation of the lesion was performed. The histopathology of the surgical enucleation specimen revealed an odontogenic keratocyst-like lesion in most parts (Fig. 2b). In a small portion, the cyst-like lesion had peripheral palisaded columnar cells with hyperchromatic nuclei, but apparent stellate reticulum-like cells were not seen (Fig. 2c); a diagnosis of an odontogenic tumor suggestive of an ameloblastoma was made. Five months after surgery, recurrence was clinically suggested, a biopsy was performed, and the diagnosis of recurrence of ameloblastoma was made. The lesion was enucleated, and the histopathology of the enucleated specimen revealed large nests or sheets of tumor cells. The morphology of this tumor was composed of an ameloblastoma-like lesion and an oral squamous cell carcinoma (OSCC)-like lesion. In the ameloblastoma-like lesion, peripheral cells were columnar with hyperchromatic nuclei arranged in a palisading pattern (Fig. 2d). Nuclear atypia and some mitotic figures were evident in both an ameloblastoma-like lesion (Fig. 2e) and an OSCC-like lesion (Fig. 2f). No relationship to the oral mucosa was noted. The Ki-67 labeling index in the OSCC-like lesion was 20% (Fig. 2g, h). Based on these findings, this tumor was considered to be an intraosseous odontogenic carcinoma derived from a precursor odontogenic keratocyst. The histopathological findings revealed characteristics of both type 1 (PIOC arising from an odontogenic cyst) and type 2B (ameloblastic carcinoma arising de novo from an ameloblastoma or odontogenic cyst) of the widely accepted Waldron and Mustoe’s traditional classification of odontogenic carcinoma [4]. Considering the atypical histopathological views of the tumor, we chose a diagnosis of exclusion, and the final diagnosis was primary intraosseous carcinoma (PIOC) with features of type 1 and type 2B as a primary intraosseous carcinoma, NOS rather than an ameloblastic carcinoma, based on the definition of the current WHO Classification of PIOC, NOS [1]. Two years and 6 months after surgery, the patient had survived without recurrence.

### Case 2

**Fig. 3:**
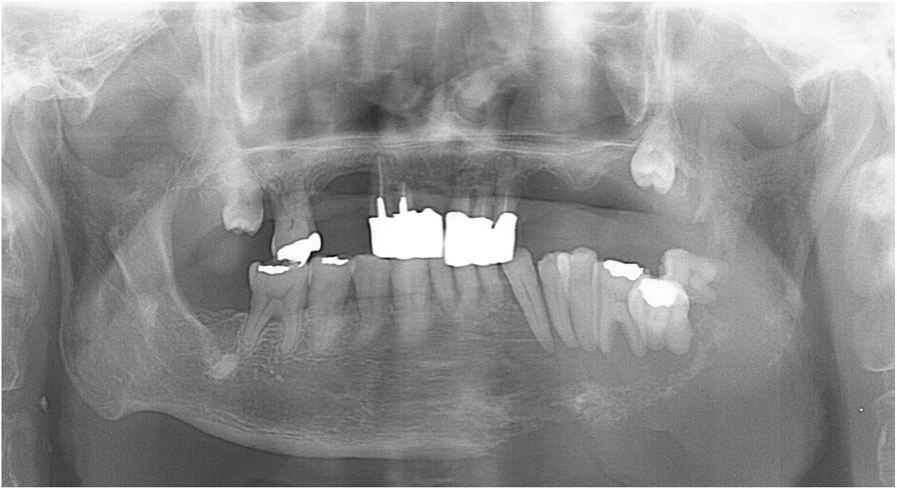
Panoramic X-ray photograph of case 2 at the first visit. Marked resorption of the left mandibular bone

**Fig. 4:**
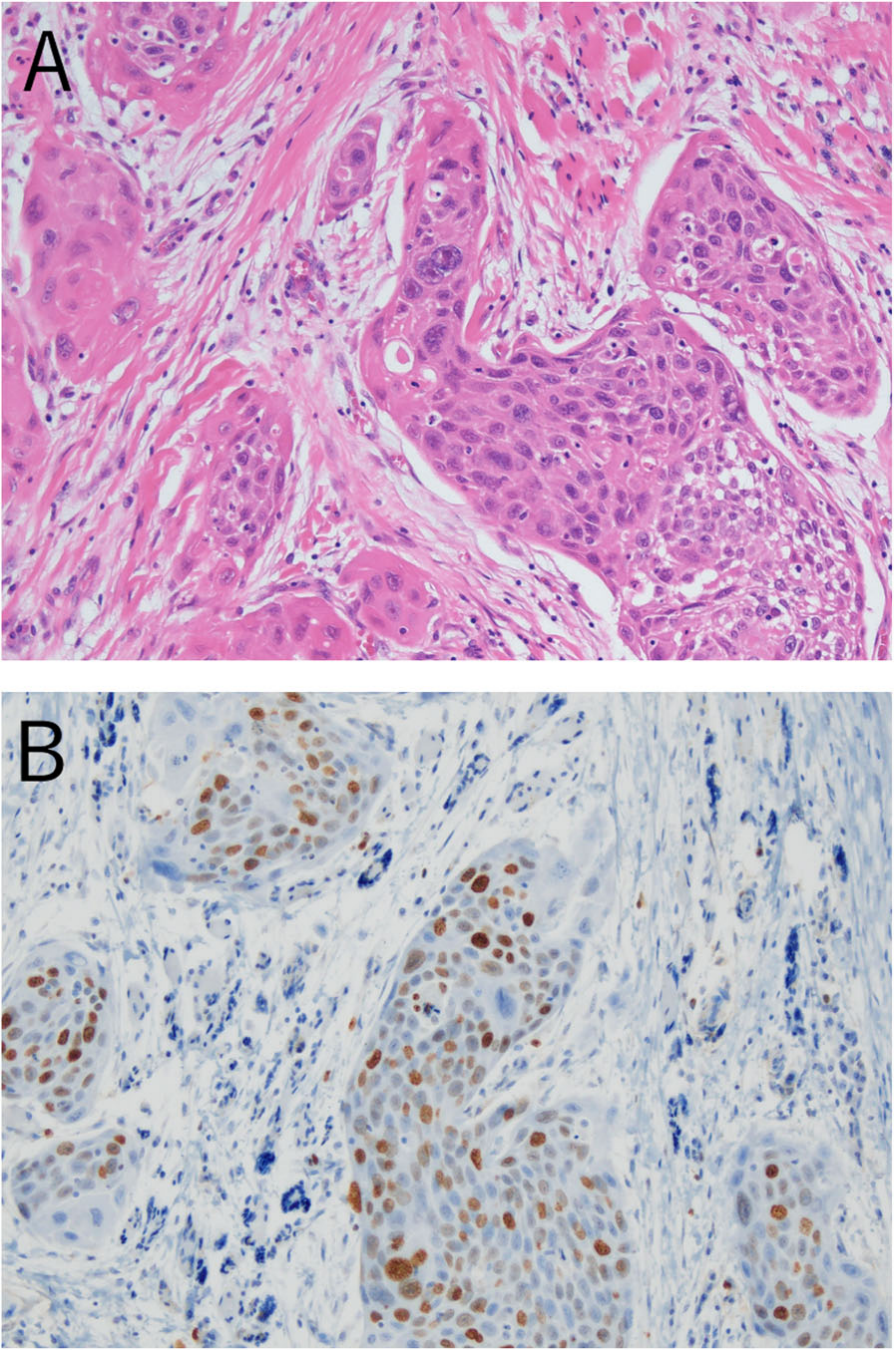
Histopathological findings of case 2. **a** Infiltration of tumor nests composed of squamous epithelial cells with atypia (H&E staining, original magnification × 400). **b** Immunohistochemistry of the tumor using Ki-67 (original magnification × 400)

A 67-year-old Japanese male presented with mobility of the front teeth and occlusal pain. He had a smoking history of 10 cigarettes a day for 47 years. Radiologically, the tumor showed marked bone resorption with progression into the left incisor region to the mandibular ramus (Fig. 3). The tumor involved the inner pterygoid and masseter muscles, and the clinical diagnosis was a malignant tumor of the mandible. Biopsy of the region was performed, and a histopathological diagnosis of squamous cell carcinoma was made. Therefore, hemimandibulectomy was performed. After surgery, postoperative chemoradiotherapy (administration of S-1 [tegafur/gimeracil/oteracil] and 60 Gy irradiation) was performed. The surgical specimen showed islands or small nests of a neoplastic squamous epithelium with mild keratinization (Fig. 4a). Nuclear atypia and pleomorphism were evident, and a high Ki-67 labeling index (40%) was noted (Fig. 4b). The lesion was intraosseous and there were no connections between the tumor and oral mucosa. The histopathology of this tumor was similar to that of OSCC. Based on the findings of the central jaw squamous cell carcinoma isolated from the oral mucosa, a final diagnosis of PIOC, NOS was made. Three years after surgery, the patient had survived without recurrence.

### NGS

**Fig. 5:**
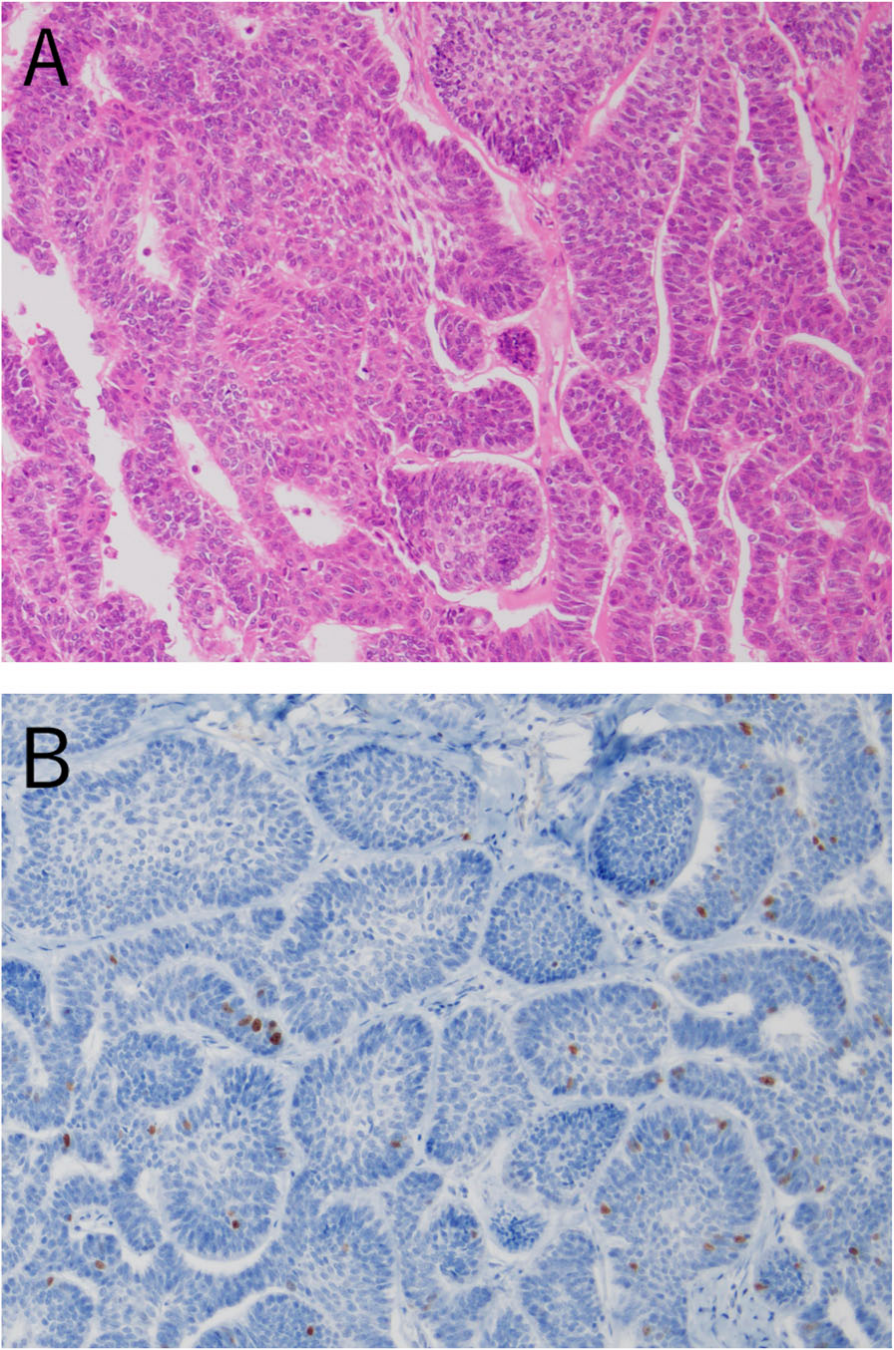
Histopathological findings of case 3. **a** Ameloblastoma composed of follicular and plexiform patterns (H&E staining, original magnification × 200). **b** Immunohistochemistry of the ameloblastoma using Ki-67 (original magnification × 200)

We analyzed mutations in hotspot regions in 50 genes commonly associated with cancer by targeted NGS in specimens from these two PIOC, NOS patients. In addition, a patient with ameloblastoma (case 3) was used as a control (Fig. 5a, b). We used formalin-fixed–paraffin-embedded specimens collected at Tokyo Medical and Dental University Dental Hospital.

DNA was extracted from 20-μm-thick sections using the QIAamp DNA FFPE Tissue Kit (Qiagen, Hilden, Germany). Library preparations were performed using the Ion AmpliSeq Library Kit 2.0 and Ion AmpliSeq Cancer Hotspot Panel v2 (Thermo Fisher Scientific, Waltham, MA, USA). The panel target’s hotspot regions included more than 2800 COSMIC mutations of 50 cancer-related genes. After library preparation, each amplicon library was quantified using the Ion Library TaqMan Quantitation Kit (Thermo Fisher Scientific) and sequenced with the Ion Proton platform and Ion 318 Chip Kit v2 (Thermo Fisher Scientific). The average read depths were approximately 770.

Data were analyzed using the Torrent Suite Software v4.2.191 (Thermo Fisher Scientific) and Ion Reporter Software v4.6 (Thermo Fisher Scientific). Read alignments were performed using the human reference genome hg19. Candidate pathogenic variants were filtered based on the number of reads in a target sequence and variant frequency in the total number of reads. Intronic, homogeneous, or synonymous variants were excluded. Mutations were analyzed using SIFT, PolyPhen, and MutationTaster and were considered to be relevant when scored as deleterious by at least two of these algorithms.

The NGS results are shown in Table 1. No mutations were observed in case 1. In contrast, missense point mutations in *TP53 Glu285Val*, *KDR Gln472His*, and *APC Pro1433Leu* were noted in case 2. The *BRAF Val600Glu* mutation was found in the patient with ameloblastoma (case 3).

**Table 1:** Summary of SNPs analyzed by NGS for the two patients with PIOC, NOS and a patient with ameloblastoma

Patient no.	1. PIOC, NOS	2. PIOC, NOS	3. Ameloblastoma
Age	28	67	32
Sex	M	M	M
Location	Left mandible	Left mandible	Left mandible
Size (mm)	75	35	45
SNP in next-generation sequencing	none	TP53 p.Glu285Val (c.854A > T)	BRAF *p*.Val600Glu (c.1799 T > A)
		KDR p.Gln472His (c.1416A > T)	
		APC p.Pro1433Leu (c.4298C > T)	

## Discussion

In the present WHO Classification of Head and Neck Tumors published in 2017 [1], odontogenic carcinomas are classified into ameloblastic carcinoma, PIOC, NOS, sclerosing odontogenic carcinoma, clear cell odontogenic carcinoma, and ghost cell odontogenic carcinoma. In contrast to other odontogenic carcinomas, PIOC, NOS does not have unique morphological features, and the name PIOC, NOS denotes a central jaw odontogenic carcinoma that cannot be categorized into the other odontogenic carcinomas.

The current WHO classification of odontogenic carcinomas added a diagnosis of exclusion, PIOC, NOS. Some PIOC, NOS cases have been shown to derive from different origins and may be further divided into multiple subcategories. Genetic alterations detected by NGS have recently provided valuable information for clarifying the oncogenesis. In the head and neck region, *BRAF* gene mutations are detected at a high frequency in ameloblastomas [3, 5, 6], and this was also confirmed not only by genetic analysis but also by immunohistochemistry using a BRAF V600E-mutant specific antibody [7–9]. We recently reported that 10 out of 11 patients with calcifying cystic odontogenic tumors (calcifying odontogenic cyst) have mutations in the *CTNNB1* gene, while 12 out of 14 patients with ameloblastoma have mutations in the *BRAF* gene [10]. We also reported a patient with ghost cell odontogenic carcinoma with a mutation in the *CTNNB1* gene, suggesting that *CTNNB1* gene mutations are one of the common features of lesions accompanied by ghost cell keratinization [11]. These findings confirmed that genetic alterations not only provide valuable information on oncogenesis but also contribute to the diagnosis and classification of odontogenic lesions. NGS may also contribute to the further study of odontogenic carcinomas; however, the greatest limitation to this research is the rarity of these lesions.

In the present study, we identified two cases of PIOC, NOS with different clinical and histopathological features and compared gene mutations using NGS. Case 1 exhibited features of both ameloblastic carcinoma and OSCC; however, *BRAF* gene mutations, which are frequently detected in ameloblastoma, were not detected. In addition, no gene mutations were identified using the Ion AmpliSeq Cancer Hotspot Panel v2. To reach a final diagnosis of PIOC, NOS in case 1, we carefully considered a differential diagnosis of ameloblastic carcinoma. Based on histopathological findings without definite features of ameloblastoma or ameloblastic carcinoma in the precursor lesion and the atypical histopathological views of the tumor, we finally chose a diagnosis of exclusion and diagnosed this PIOC with features of type 1 and type 2B as PIOC, NOS, following the current WHO classification of odontogenic carcinomas. Although PIOC, NOS includes tumors arising in odontogenic precursor lesions, reports of odontogenic tumors derived from precursor lesions are very limited, and most of them have features of OSCC [1]. Some cases of ameloblastoma arising in the wall of dentigerous cysts have been reported [12, 13]. Except for dentigerous cysts, only a few cases of ameloblastoma arising in glandular odontogenic cysts have been reported [14]. Case 1 in this study is an extremely rare report of PIOC, NOS with features of both ameloblastic carcinoma and squamous cell carcinoma arising in a precursor cyst. This may also be considered a case of ameloblastic carcinoma arising in a precursor cyst, but the sole histopathological criterion is PIOC, NOS.

Case 1 did not have any gene mutations, whereas case 2, which had features of OSCC, had mutations in the *TP53*, *KDR*, and *APC* genes. *TP53 Glu285Val*, which is located in the DNA-binding domain at codon 285 (H2 α-helix) of *TP53*, resulted in a glutamic acid to valine substitution. Russell-Swetek et al. reported that functional analyses of *TP53* Glu285Val revealed significant defects in its ability to regulate promoter activity, suppress tumor cell growth, and trigger apoptosis, and *TP53* Glu285Val efficiently functions as a dominant negative regulator that neutralizes wild-type p53 activity [15]. *TP53 Glu285Val* was reported in a pediatric case of adrenocortical carcinoma and choroid plexus carcinoma [15]. There have been no reports on mutations in OSCC; however, *TP53 Arg282Trp* was detected in a case of OSCC that was located in the same DNA-binding domain of *TP53 Glu285Val* [16]. The *KDR* gene recognizes vascular endothelial growth factor receptor-2, and the *KDR Gln472His* mutation has been detected in lung and prostate cancers [17, 18]. *APC Pro1433Leu* was also detected in renal cell carcinomas [19], and these results suggest that the tumor in case 2 arose from an odontogenic epithelium by mutations found in other cancers.

We previously reported that BRAF mutations were present in more than 85% of ameloblastoma cases [10]. Although no definitive data were present, case 1 may have been a tumor other than ameloblastoma, and oncogenesis in this case may have been attributed to mutations other than those in Ion AmpliSeq Cancer Hotspot Panel v2 or to other gene alterations, such as translocations. In contrast, PIOC, NOS, with many morphological similarities to OSCC, has similar gene mutations to OSCC. These results suggest that these two PIOC, NOS cases belong to different subcategories. However, its low incidence leads to difficulties in performing a systematic analysis that includes many cases of these odontogenic carcinomas, which can only be done with a review of the literature [20].

## Conclusion

We reported an extremely rare case of PIOC, NOS with features of both ameloblastic carcinoma and OSCC arising in a precursor cyst and compared this atypical case to a typical case of PIOC, NOS with features of OSCC. The accumulation of data on a small number of cases is essential to improve our understanding of PIOC, NOS, and the present results suggest the importance of obtaining genetic information as well.

## Data Availability

The datasets used and/or analyzed during the current study are available from the corresponding author upon reasonable request.

## Abbreviations

NGS: Next-generation sequencing
OSCC: Oral squamous cell carcinoma
PIOC: Primary intraosseous carcinoma
